# Short- and long-term effects of Covid-19 pandemic on health care system for individuals with eating disorders

**DOI:** 10.3389/fpsyt.2024.1360529

**Published:** 2024-03-14

**Authors:** Margherita Boltri, Federico Brusa, Emanuela Apicella, Leonardo Mendolicchio

**Affiliations:** I.R.C.C.S. Istituto Auxologico Italiano, Experimental Laboratory for Metabolic Neurosciences Research, Piancavallo, VCO, Italy

**Keywords:** eating disorders, COVID-19 pandemic, healthcare system, short-and long-term effects, systematic literature review

## Abstract

**Introduction:**

The Covid-19 pandemic and its consequences have negatively impacted the incidence of EDs, determining a substantial burden on patients, caregivers and healthcare systems world-wide. This literature review aims to investigate the short- and long-term effects of the pandemic on care provider systems, exploring the possibility of “rethinking” ED care programs.

**Methods:**

Records were systematically (following the PRISMA guidelines) identified through PubMed, Google Scholar and Scopus searching.

**Results:**

The Covid-19 pandemic led to an abrupt and substantial increase in pediatric and adolescent ED visits and hospital admissions. Despite a decline in the second-year post-onset, absolute visit volumes remained elevated relative to pre-pandemic levels. Barriers to access specialist ED care have emerged, including socio-economic status and a lack of public outpatient services. Consequently, this situation has prompted healthcare providers to explore innovative bridge plans and multidisciplinary telehealth solutions to face such challenges.

**Discussion:**

Challenges in insurance shifts, treatment disruptions and discharge planning underscore the need for comprehensive strategies in ED care. Overall, our findings highlight the importance of adopting multidisciplinary approaches, implementing location-specific plans, and integrating telehealth to effectively address the evolving challenges posed by the pandemic and enhance the efficiency of ED specialist care programs.

## Introduction

The emergence of the global Covid-19 pandemic in recent years has not only strained healthcare systems and resources but has also had far-reaching effects on various aspects of medical care, extending well beyond the immediate realm of viral infection (1). Among the many areas impacted by the pandemic, the provision of care for patients with Eating Disorders (ED; 2) has emerged as a significant concern (3). The Covid-19 pandemic has presented a multitude of challenges that have reverberated across the entire spectrum of healthcare, affecting patients, caregivers, and healthcare systems worldwide (4).

Research indicates a concerning surge in hospital admission rates to specialized ED units in various European countries, particularly Italy, Spain, Sweden, and France (5). Notably, the reported waiting times for accessing care nearly doubled in these countries, coinciding with clinicians’ observations of exacerbated ED symptoms and general psychopathology. A recent systematic review of hospital admissions during the pandemic revealed an average 48% increase in ED admissions (pre = 591, post = 876) compared to previous periods. Additionally, when comparing pediatric admissions to those of adults, there was an average 83% increase in pediatric admissions, while adult admissions saw an average 16% rise (6).

The symptomatology of eating disorders has undergone subtle changes in the wake of the Covid-19 pandemic. Research conducted across Europe and other global regions, including Australia and Canada (7, 8), has identified a significant increase in restrictive eating, exercise, and social media use. Such factors, as well as social isolation and disruptions to daily routines, but also treatment interruptions appear to have worsened the ED risk (9, 10) and increased the likelihood of relapse in individuals with pre-existing EDs (11, 12). Moreover, numerous studies have reported elevated levels of anxiety and depression during the pandemic. These increases in eating disorder symptoms were observed across all groups of Anorexia Nervosa (AN; 2), Bulimia Nervosa (BN; 2), Binge-Eating Disorder (BED; 2), and Other Specified Feeding or Eating Disorder (OSFED; 2) patients (6). As for the post-lockdown period, some studies have indicated improvements in ED symptoms (13), while others have reported worsening symptoms post-lockdown, such as increased binge eating (14). By definition, ED encompass a wide range of conditions, which are intricate and multifaceted, requiring a structured, often long-term approach to treatment and care. They not only impose physical debilitation but also have profound psychological and emotional impacts on those affected, often necessitating intensive support and intervention. The pandemic has compounded the challenges associated with managing these disorders, underscoring the need for a comprehensive examination of care provision. For individuals with EDs, annual healthcare costs are 48% higher than those of the general population (15). Moreover, due to Covid-19-related social distancing guidelines and restrictions, traditional face-to-face interactions and access to care have been limited, prompting clinicians to transition to the use of telehealth platforms in the context of mental health care (16). Notably, studies have introduced a range of digital health interventions, including web-based, mobile therapy, and virtual reality, in the delivery of care for eating disorders (17). However, approximately 50% of patients interrupted some treatment during the pandemic, and around 30% perceived disruptions in their treatment (10). These factors and abrupt changes in care delivery services may have contributed to the exacerbation of ED symptoms.

In this context, it is imperative to gain a deep understanding of the relationship between care changes during and after the pandemic, exploring the strategies implemented by specialized ED units to address these issues and the integration of traditional care with telehealth. This understanding is essential for learning from the pandemic’s impact and developing updated guidelines for ED care programs.

This systematic literature review seeks to delve into the short- and long-term effects of the pandemic on care provider systems within the context of EDs. By doing so, we aim to explore the possibility of rethinking and reconfiguring the existing ED care programs, with a view to enhancing their effectiveness and adaptability to the evolving healthcare landscape.

## Methods

### Study design

The following systematic review was completed in accordance with Preferred Reporting Items for Systematic Reviews and Meta-Analyses (PRISMA; 18).

### Inclusion and exclusion criteria

The inclusion criteria were defined as follows: (1) scientific articles published in either English or Italian; (2) articles published from March 2020, coinciding with the onset of the Covid-19 pandemic, onwards; (3) studies addressing the effects of Covid-19 on the healthcare system and the management of individuals with eating disorders; (4) studies covering all age groups - this encompasses research involving pediatric, adolescent and adult population; (5) studies must be based on a clinical environment specialized in the treatment of ED or within the psychiatric community/hospital setting – this ensures a focused investigation into the clinical management and treatment of ED; (6) articles must employ established diagnostic criteria for ED, notably, the International Classification of Diseases 10th Revision (ICD-10) or the Diagnostic and Statistical Manual of Mental Disorders Fourth or Fifth Edition (DSM-IV or DSM5-5) to diagnose eating disorders; (7) articles should involve clinical populations diagnosed with ED – this excludes studies focusing solely on non-clinical populations, such as students or individuals without a ED diagnosis, even if they exhibit symptoms. Articles that do not specifically investigate the effects of Covid-19 on the healthcare system and the management of ED will not be considered.

### Search procedures

A search was conducted in October 2023 and updated in February 2024. PubMed, Scopus, and Google Scholar databases were searched, and the following keywords with respect to each database search strategy were applied: “Covid-19” AND “Eating Disorders” AND “healthcare system” AND “access to care”. The search strategy was tailored to each database and included Boolean operators (AND, OR) to combine search terms, as well as additional search filters to retrieve relevant literature. The exact search strings used for each database are provided in **Table 1**.

**Table 1:** Search terms and strategy.

**covid 19**	(“COVID-19” OR “COVID-19”[MeSH Terms] OR “COVID-19 Vaccines” OR “COVID-19 Vaccines”[MeSH Terms] OR “COVID-19 serotherapy” OR “COVID-19 serotherapy”[Supplementary Concept] OR “COVID-19 Nucleic Acid Testing” OR “covid-19 nucleic acid testing”[MeSH Terms] OR “COVID-19 Serological Testing” OR “covid-19 serological testing”[MeSH Terms] OR “COVID-19 Testing” OR “covid-19 testing”[MeSH Terms] OR “SARS-CoV-2” OR “sars-cov-2”[MeSH Terms] OR “Severe Acute Respiratory Syndrome Coronavirus 2” OR “NCOV” OR “2019 NCOV” OR ((“coronavirus”[MeSH Terms] OR “coronavirus” OR “COV”)
**eating disorders**	“feeding and eating disorders”[MeSH Terms] OR (“feeding”[All Fields] AND “eating”[All Fields] AND “disorders”[All Fields]) OR “feeding and eating disorders”[All Fields] OR (“eating”[All Fields] AND “disorders”[All Fields]) OR “eating disorders”[All Fields] health care system: “delivery of health care”[MeSH Terms] OR (“delivery”[All Fields] AND “health”[All Fields] AND “care”[All Fields]) OR “delivery of health care”[All Fields] OR (“health”[All Fields] AND “care”[All Fields] AND “system”[All Fields]) OR “health care system”[All Fields]
**access to care**	“health services accessibility”[MeSH Terms] OR (“health”[All Fields] AND “services”[All Fields] AND “accessibility”[All Fields]) OR “health services accessibility”[All Fields] OR (“access”[All Fields] AND “care”[All Fields]) OR “access to care”[All Fields]

### Study selection

**Figure 1:**
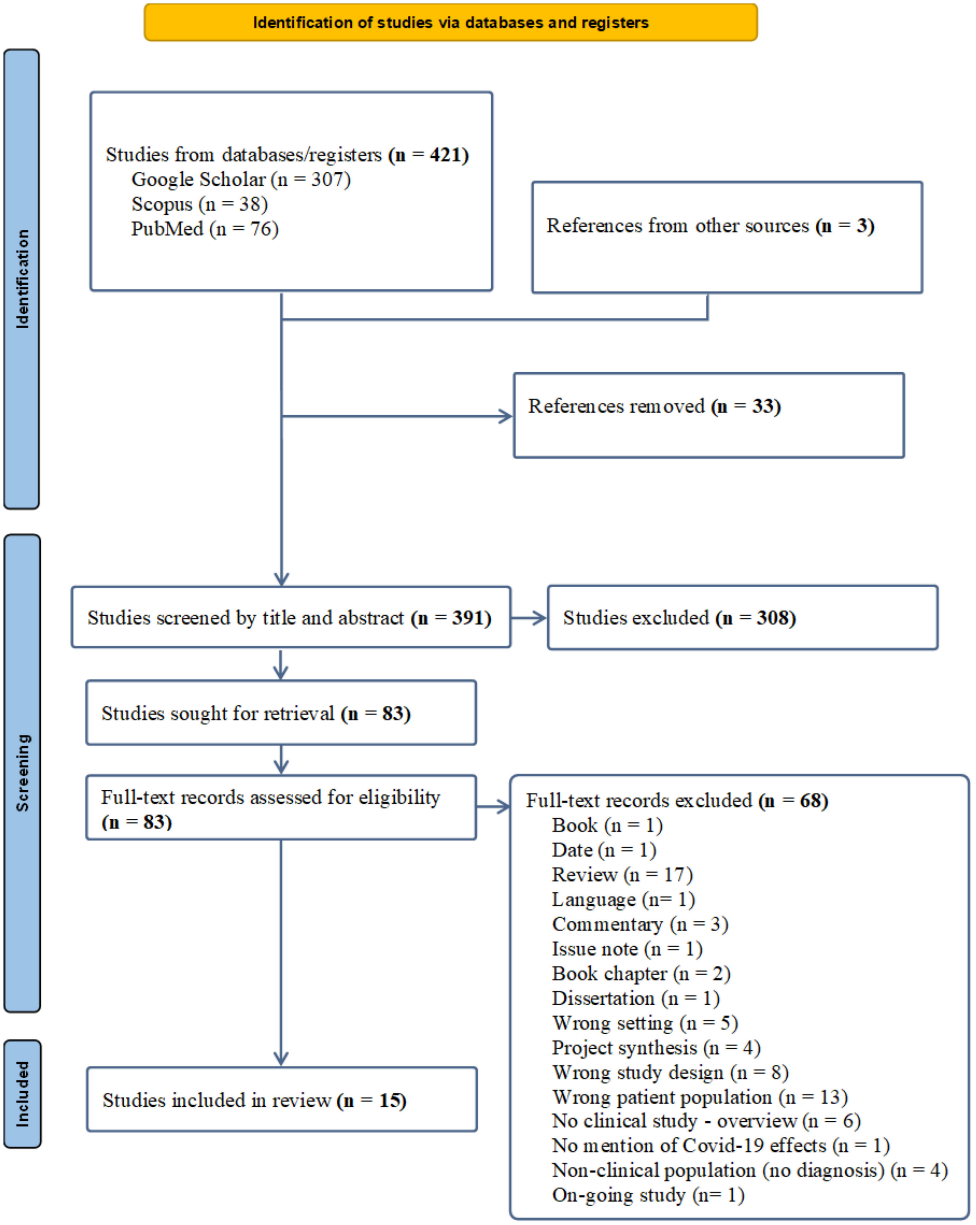
PRISMA 2020 flow diagram for systematic reviews (18).

All studies were required to be published in a peer-reviewed journal and written in English or Italian. We used the PICO approach to guide the search strategy for this review. The reference lists of eligible studies were manually screened for additional literature not otherwise captured. Two investigators (MB and FB) independently and blindly performed the searches. Articles were initially screened by title and abstract. The remaining articles were further scrutinized by full-text review to determine eligibility for inclusion. Discrepancies between reviewers were resolved through discussion and consensus. The exact search strategy is presented in **Figure 1**.

### Validity assessment

The validity of the included studies was examined against a detailed set of criteria, which included up to 14 questions derived from the National Institutes of Health Study Quality Assessment Tools 69. If a study sufficiently met a question’s criteria it was coded a “Yes”, otherwise it was coded a “No”, “Not Reported”, “Cannot Determine” or “Not Applicable”. Following criteria guidelines, studies were then rated as “Good”, “Fair”, or “Poor” by two independent authors (MB and FB), and studies were not penalized for questions deemed “Not Applicable”.

### Bias assessment

Per guidelines, two independent authors (MB and FB) assessed methodology against criteria assessing six types of bias: selection, performance, detection, attrition, reporting, or other, according to our study quality assessment tools. Discrepancies in bias assessment between reviewers were resolved through discussion and consensus.

## Results

### Database search

The database search, supplemented by references from other sources (n= 3), initially identified 421 records, with 391 remaining after duplicates were removed taking advantage of the Mendeley Reference Manager. Following screening by title and abstracts, 83 studies were identified as potentially relevant. At this stage, identified papers were screened manually to identify and omit non-relevant studies, that did not meet the eligibility criteria (for reasons of exclusion, please refer to **Figure 1**).

### Overview of included studies

Fifteen studies investigating the short- and long-term effects of the Covid-19 pandemic on care provider systems within the context of EDs were identified in the literature and summarized in **Table 2**. A full consensus was obtained between the two independent studies search and selection. Nine studies collected data from monocentric cohorts in USA and Australian services, while six studies were multicentric, and collected data from European, USA and Canadian clinical centers and hospital departments.

**Table 2:** Monocentric and multicentric studies investigating effects of **the** Covid-19 pandemic **on** ED care provider systems.

Authors	Country	Study design	N	Age M (SD)	N of ED centers involved	Diagnosis	Period of analysis	Main results	Short term effects	Long-term effects
Monocentric studies										
Feldman et al., 2022 (19)	USA	Retrospective study	71	14.6 (2.12)	1 (pediatric hospital)	AN-BP = 53 AN-atypical= 3 ARFID= 9 OSED= 3 OSFD= 3	Jan 2017 – July 2021	188% increase in ED related hospital admissions (pre-Covid-19: 0.816 patients per month; during Covid-19: 2.35 patients per month). 2.9 times more hospital admissions related to medical stabilization of patients with EDs than in the 3 years prior. Comorbid anxiety and/or depression during Covid than pre-Covid (χ^2^ = 8.404, *p* = .004) with a 97% increase.	Direct transfers to the recommended higher level of care after medical stabilization significantly decreased (χ^2^ = 6.06, *p* = .014). Outpatient bridge plans significantly increased (χ^2^ = 4.27, *p* = .039) during Covid, with 42.5% of patients needing bridge plans during Covid compared to 19.3% of patients pre-Covid. Average length of hospital stay was found to slightly increase during Covid (M = 14.8, SD = 10.9) as opposed to pre-Covid (M = 12.6, SD = 7.43), although this change was found to be non-significant (F[69] = 2.49, p = .341).	Significant increase in private insurance patients and a decrease in Medicaid patients during Covid-19 when compared to pre-pandemic (χ^2^ = 4.01, *p* = .045). Increased demand for inpatient ED medical stabilization and post-discharge higher level of care → bridge plans as a possible solution Need for greater access to multidisciplinary care at the outpatient level
Hellner et al., 2020 (20)	USA	Beta trial	2	Age: 15 and 20	1 (private clinical center)	AN-atypical=1 AN=1	April – May 2020	Patients gained 1 pound per week during the trial (4 weeks treatment). Marked decrease in ED symptoms. Engagement and satisfaction were high	Virtually delivered FBT models was positively perceived by carers and patients	No follow-up measures
Ibeziako et al., 2022 (21)	USA	Retrospective study	3799	Age range 4-18	1 (pediatric hospital- emergency department)	ED = 385	March 2019 – February 2021	Significant increase in ED presentation from 2019-2020 compared to 2020-2021 → from 6.9% (n= 139) to 13.8% (n= 246) presentations (χ^2^ = 50.12, *p*<.001)	Admissions for children declined (22.0% vs 16.2%) and adolescents increased (78.0% vs 83.8%, *p* <.001). Length of admission (2.5 vs 5.5 days, *p* <.001) and length of boarding (2.1 vs 4.6 days, *p* <.001) more than doubled during the pandemic year. 71.5% of pediatric patients presenting with mental-health issues (n = 1272) boarded in the ED unit	Decrease in patients with public insurance (46.0% vs 41.7%, *p* = .008) and consequent increase in private insurance There was a significant difference in the pre-pandemic and pandemic phases (*b* = 1.941, t = 3.673, *p*= .002), with a steep positive linear slope representing 68.2% of the increase of ED (which has continued in 2021)
Levinson et al., 2021 (22)	USA	Between-groups interventional study	93	24.87 (8.35)	1 (intensive outpatient program)	AN= 40 BN= 10 OSFED= 32 BED= 9 ARFID= 2	March 2018 – Jan 2021	Parental criticisms such that those in the in-person (M = 11.58; SD = 8.82) condition were slightly more concerned about criticism than those in the telehealth condition (M = 8.82; SD = 3.90), t(91) = 2.76, *p* = .003. Significant difference on parental expectations, such that those in the in-person (M = 15.40; SD = 4.76) condition were slightly more concerned about parental expectations than those in the telehealth condition (M = 12.36, SD = 3.98), t(91) = 3.03, p = .002)	ED symptoms decreased similarly regardless of condition (*p* = .243) BMI increased similarly regardless of condition (*p* = .058).	No significant differences were found between participants receiving telehealth treatment versus in-person format. Telehealth transition due to the Covid-19 pandemic is effective for treatment delivery → multi-disciplinary team-based approaches via telehealth
Otto et al., 2021 (23)	USA	Retrospective study	248	15.2 (2.6)	1 (pediatric hospital)	AN= 177 Atypical AN= 43 ARFID= 20 UED= 6 OSED= 2	March 2017- 2021	The majority of patients, both before and during the pandemic, were female and white Counts of ED-related admissions per month increased throughout the Covid-19 era, with the highest counts observed near the end of the study period, 9 to 12 months after the pandemic began. It is not clear whether this increase is a function of delayed care and/or an increase in incident cases	Counts of admissions per month then increased significantly over time during the Covid-19 pandemic β1 + β3 = 1.58 [95% CI: 1.25 to 1.91]; p<.001. The total number of admissions between April 1, 2020, and March 31, 2021, (n = 125) was more than double (123% increase) the mean number of admissions for the same time frame (April 1 through March 31) for the previous 3 years (M = 56).	Significant change in the distribution of insurance types during the Covid-19 pandemic (*p* = .005), and the proportion of patients with public insurance decreased significantly (*p*= .02). Restrictions on in-person outpatient clinic appointments ended in June 2020, whereas inpatient admissions continued to increase significantly through March 2021, suggesting these brief limitations in in-person outpatient appointments were not a significant driver of the increase in admissions
(24)	USA	Pilot trial	12	Age range 2-7.5	1 (ED outpatient program)	ARFID= 12	2020 (not specified)	Participants who received outpatient follow-up in-clinic or via telehealth met an equivalent percentage of their goals over time	Participants in the in clinic and telehealth groups met 92% and 100% of their goals	Results indicate that telehealth services are just as effective as in-clinic services for meeting meaningful goals and maintaining appropriate participant and caregiver behavior during outpatient follow-up
(25)	Australia	Comparative observational study	25	24.2 (7.62)	1 (ED specialist outpatient public unit)	AN= 48 BN= 20 OSFED= 28 UFED= 4	March-April 2020	Despite lockdown and integration of telehealth, substantial improvements on all outcomes were reported	Switching to telehealth was not associated with a notable deterioration in ED symptoms Reductions for ED symptoms and clinical impairment were observed Mood (depression and anxiety) also improved BMI returned from underweight range to the typical weight range (*p* <.001, *d* = 2.93)	Most patients (71%, n = 12) rated telehealth as being “as good as” or “better than “face-to-face Quality of therapeutic relationship during telehealth was rated “as good as usual” (88%, n=15) compared to face-to-face
(10)	USA	Retrospective study	73	19.1 (3)	1 (children hospital – outpatient program)	AAN = 85% Other ED (BED, BN, AIRFID)= 15%	June 2017 – February 2020	81% endorsed increased intrusive ED thoughts and behaviors as a result of the COVID-19 45% of the participants reported engaging in restrictive/compensatory/or binging behaviors frequently or daily	Perceived treatment disruption was associated with lower quality of care (*p* = 0.004) Of those with telehealth access (n = 64), 59% found care as good as usual	Perceived treatment disruption was associated with higher odds of intrusive ED thoughts (aOR = 2.63; 95% CI: 0.56–12.3) and increased ED behaviors (aOR = 1.98; 95% CI: 0.63–6.19). Maintained access to ED care had higher odds of intrusive ED thoughts (aOR = 5.32; 95% CI: 0.72–39.6) but lower odds of increased ED behaviors (aOR = 0.67; 95% CI: 0.11–4.20).
Springall et al., 2021 (12)	Australia	Retrospective chart review	457	15	1 (specialist ED program in pediatric hospital)	ED= 161	2017-2020	Most presentations were new ED diagnoses (>90% each year) and most were female (>80% each year). In 2020, ED presentations far exceeded mean number of presentations per year from 2017-2019 (N = 98.7, *p* = 0.01) Among the primary reasons reported for the ED onset: social isolation and loneliness (32.3%), change in normal routines and subsequent lack of motivation (25.6%), cessation of community sport, boredom, and minimal distraction from ED thoughts A small percentage (3%) reported reduced food availability as a factor.	Between February and April 2020 there was a decrease in new diagnoses of AN and atypical AN Starting from May, there was an increase in the number of cases presenting each month, and from June onward, the number of cases exceeded those recorded for the same month in the previous three years For 40.4% of patients assessed in 2020, the onset of ED behaviors coincided with the Covid-19 lockdown. Additionally, 12.8% of patients experienced a relapse in progress during the Covid-19 lockdown.	The peak of new cases of restrictive ED occurred in August 2020, followed by a period of stabilization and a slight decrease starting in September The proportion of AN and atypical AN patients admitted to hospital only slightly increased from 2017- 2020 (55.9 to 62.4%) No significant changes in the incidence of purging behavior and suicidal or self-harm ideation were reported between years (p = 0.85)
Multicentric studies										
Milliren et al., 2023 (26)	USA	Retrospective study	ED visits= 8010 Inpatient admissions = 9302	15.5 (2.3)	38 (pediatric hospitals)	ED visits: AN = 5082 ARFID = 888 BN or BED = 267 OSED= 298 UED= 1475 ED inpatient: AN= 6587 ARFID= 1136 BN or BED= 220 OSED= 274 UED= 905	Jan 2018 – June 2022	Following an initial drop, emergency department visits surged in the first year of the pandemic, plateauing in the second year at levels higher than pre-pandemic. Although stabilized, the ED volumes remained elevated compared to pre-pandemic periods → in the pre–Covid-19 period, the mean number of ED-related admissions per hospital was 93.9 (SD = 116.6; range 3–514) compared with 150.8 (SD = 192.9; range 5–992) post–Covid-19 onset. Despite a decline in the second year, absolute volumes were still elevated relative to pre-pandemic levels 27 months after pandemic onset, particularly for ED visits.	A total of 95% of hospitals had higher aggregate raw volumes and higher average monthly visit volumes in the post-onset period. No significant difference in percentage of ED visits that resulted in inpatient admission pre– versus post–Covid-19 onset (n = 2057, 73.7% versus n= 5 3773, 72.3%, respectively; *p* = .20). After the onset of the pandemic, a higher proportion of inpatient admissions for EDs were among patients of adolescent age (14–17 years), female sex, white race, non-Hispanic, privately insured, and from higher-median income zip codes	Post–Covid-19 onset, there was an immediate decline in the number of visits (β= -48.7; 95% CI: -79.1 to -18.2; *p*= .002), followed by a significant increase over time (β= 12.9 per month; 95% CI: 9.2–16.6; *p* <.001) through the first year in March 2021. In the second year post-onset through the end of the study period (April 2021–June 2022), visit volume decreased over time (β= -6.3 per month; 95% CI: -9.0 to -3.5; *p*<.001). More diagnoses of AN after the onset of Covid-19 ED inpatient admissions were slightly longer post-onset of the pandemic, which, in combination with increased volume, resulted in a nearly 66% increase in monthly average cumulative bed-days
(27)	Italy	Multicentered multilevel modeling	312	AN= 26.92 (10.28) Other ED = 32.24 (13.53)	16 (specialist ED units)	AN= 179 BN= 63 BED= 48 OSFED= 22	June 2020	Perceived therapeutic relationship quality showed significant negative effects on general psychopathology (β=−0.16; *p*=0.007) and eating-related psychopathology worsening (β=−0.22; *p*=0.001). Heightened isolation and fear of contagion had significant positive effects on general psychopathology worsening (β = 0.22; *p* = 0.002 and β = 0.16; *p* = 0.003, respectively) and eating-related psychopathology worsening (β=0.22; *p <*0.001 and β=0.17; *p*=0.002, respectively).	Not the type of treatment provided during the Covid-19 lockdown period but a higher quality of perceived therapeutic relationship was associated with a lower increase of psychopathology severity 2/3 of the sample had direct access to care during the Covid-19 lockdown, while the majority of the remaining sample was provided with a telehealth treatment	Perceived quality of therapeutic relationship (and not the type of intervention) was found as a putative protective factor towards psychopathology worsening in case of similar stressful events (like Covid-19 lockdown)
Monteleone et al., 2023 (28)	European multicentered study (Italy, France, Spain, Czech Republic, Germany, Poland, UK)	Multicentered cross-sectional study	409	26.6 (11.2)	11 (specialist ED units – inpatient, outpatient and day-hospital)	AN= 89 BN= 41 BED= 66	Sep – Oct 2020	Longer time between the onset of symptoms of the current ED episode and access to a specialist ED unit was associated with higher age (*p*<.001), general psychiatric symptoms (*p*= .01) and low social class (*p*= .008). The suggestion to seek care that promoted being seen at the specialist ED unit came from patients themselves (42%) or family (40.3%) in most of the cases. Psychiatrists, general practitioners and psychologists were the most common health professionals who either started the specialist pathway to ED care or directly referred to ED specialized units	Pathway to care: Median time elapsing between the onset of symptoms of the current ED episode and the access to a specialist ED unit was 2 years (Min=0; Max=36). The average number of health professionals included in the specialist pathway for ED care was 2 (Min=0; Max=10). Most of the participants (92%) did not directly access the specialist ED unit usually saw two other health professionals first Country-related differences: Spain, Czech Republic, and Germany were associated with delayed access to ED units in comparison to Italy, while UK had an earlier access.	Not investigated
Hartman-Munick et al., 2022 (29)	USA	Observational case series	3100	Age range (5-26)	14 (hospital-based adolescent medicine center) 1 (nonhospital-based center)	ED (specific diagnoses were not reported)	Jan 2018 – Dec 2021	A significant increase in both inpatient and outpatient ED volume after onset of the pandemic that surpassed pre-pandemic patient care trends (particularly through the first year)	Inpatient admissions: The aggregate number of ED admissions across all sites reached 163 in December 2020, peaking at 208 in April 2021 with a mean of 181 per month through the last 8 months of 2021. Pooled results accounting for site showed that prior to the pandemic, inpatient ED-related admission volume was increasing by a mean of 0.7% (95% CI, 0.2%-1.3%) each month. In April 2020, there was a small, although not significant, immediate 14.6% decline (95% CI, −29.2% to 2.9%) on average in inpatient ED admissions followed by a significant 7.2% (95% CI, 4.8%-9.7%) per-month increase in volume on average through April 2021. Beginning in May 2021, there was an immediate, nonsignificant 15.5% decline (95% CI, −30.2% to 2.3%) in ED-related admissions followed by a significant decrease over time of 3.6% (95% CI, −6.0% to −1.1%) per month on average through December 2021. Outpatient admissions: The aggregate number of assessments across all sites was 274 in December 2020, peaking at 425 in March 2021, and there was a mean of 376 per month through the last 8 months of 2021 Immediately post-pandemic onset, outpatient ED assessment volume declined by 39.7% (95% CI, −50.4% to −26.7%) but then increased significantly over time by a mean of 8.1% (95% CI, 5.3%-11.1%) per month through April 2021. Starting in May 2021, there was a significant immediate decline of 22.0% (95% CI, −36.4% to −4.4%) in the number of assessments followed by a nonsignificant decrease over time of a mean of 1.5% (95% CI, −3.6% to 0.7%) per month through December 2021	
Toulany et al., 2022 (30)	Canada	Cross-sectional study	ED visits= 1542 ED hosp= 2300	10.1(4.3) Age range (3-17)	Data from Ontario public hospitals (at least 140)	ED (no specific n. for each diagnosis was reported)	Jan 2017 – Dec 2020	Significant increase in emergency department visits and hospitalizations for ED among children and adolescents (66% and 37%, respectively) after the onset of the Covid-19 pandemic compared with pre-pandemic expected rates	Post-pandemic onset, acute care visits peaked in October 2020, with ED visits reaching an annualized rate of 34.6 per 100,000 and hospitalizations at 43.2 per 100,000. ED visits continued significantly above the three-year pre-pandemic average until December 26, 2020. Aged 3-13: - ED visit rates were lower than expected in April and May before returning to expected levels in June, 2020 (aRR = 1.04, 95% CI = .66-1.63). ED visit rates continued to be higher than expected before peaking in December, 2020 (aRR= 2.40, 95% CI = 2.08-2.77) - Hospitalizations were higher than expected after the onset of the pandemic (aRR = 1.29, 95% CI= 1.03-1.61) Aged 14-17: - ED visits for adolescents were higher than expected for all the months after the onset of the pandemic - Hospitalizations for these adolescents were higher than expected (aRR= 1.43, 95% CI= 1.33-1.53).	
Vyver et al. (31)	Canada	Hospital-based study	ACH= 890 SickKids= 208	ACH= 14.0 (1.96) SickKids= 14.2 (1.78)	2 (children hospitals)	AN= 791	March 2014 – March 2021	649 (72.9%) children and adolescents from SickKids and 142 (68.3%) from ACH had the most responsible diagnosis of AN	Similar patterns were seen in both hospitals, suggesting an increase in hospitalizations (63% increase at SickKids and 132% increase at ACH) after the pandemic onset and during the first year of Covid-19 At each site, the final three months (December, 2020 to March, 2021) had lower counts, suggesting a nonlinear pattern	

AAN, atypical anorexia nervosa; ACH, Alberta Children’s Hospital; AN, anoressia nervosa; AN-BP, anoressia nervosa binge-purging; ARFID, avoidant restrictive food intake disorder; aOR, adjusted odd-ratio; aRR, adjusted relative rate; BN, bulimia nervosa; BED, binge eating disorder; CI, confidence interval; ED, eating disorder; hosp, hospitalizations; M, mean; SD, standard deviation; OSFED, other specified eating disorder; SickKids, Hospital for Sick Children; UED, unspecified eating disorder; UFED, unspecified feeding and eating disorder.

### Monocentric studies

Feldman et al. (19), Ibeziako et al. (21), Otto et al. (23), Spigel et al. (10) and Springall et al. (12) investigated admission patterns and trend of patients with EDs in adult clinical units and pediatrics hospitals in Australia and across the USA.

In the retrospective chart review conducted by Feldman et al. (19) with 70 adolescents and young adults aged between 10 and 21 years, aiming to both quantify the increase in medical stabilization secondary to restrictive EDs and identify trends of patients requiring hospitalization before and during the Covid-19 pandemic (from January 2017 and July 2021) at a children’s hospital in the southeastern United States (US), findings report a worrying 188% increase in ED related hospital admissions (pre-Covid-19: 0.816 patients per month; during Covid-19: 2.35 patients per month), resulting in 2.9 times more hospital admissions related to medical stabilization of patients with EDs than in the 3 years prior. Beside somatic complications, results have shown a 97% increase in comorbid anxiety and/or depression during pandemic times. Despite the increase in number of hospitalizations, direct transfers to the recommended higher level of care after medical stabilization significantly decreased (χ^2^ = 6.06, *p* = .014). Patients during the pandemic were less likely to directly transition to specialized ED care services for treatment, whereas outpatients bridge plans significantly increased (χ^2^ = 4.27, *p* = .039). There was also a significant increase in private insurance patients during Covid-19 when compared to pre-pandemic (χ^2^ = 4.01, *p* = .045). Similarly, Ibeziako et al. (21) conducted a retrospective chart review in a children hospital emergency department to compare psychiatric diagnoses and boarding between March 2019 and February 2021. Among 3799 children and adolescents aged between 4 and 18, 385 of them suffered from EDs. Findings suggest a significant increase in ED presentation from 2019-2020 compared to 2020-2021, from 6.9% (n= 139) to 13.8% (n= 246) presentations (χ^2^ = 50.12, *p*<.001). Of all the pediatric patients who presented with mental health-related complaints during the pandemic, 71.5% boarded in the ED and/or inpatient units for >1 day and 50.4% experienced extended boarding periods of >2 days awaiting placement, compared with 56.9% and 30.2%, respectively, during the pre-pandemic year. Moreover, length of admission (2.5 vs 5.5 days, *p* <.001) and length of boarding (2.1 vs 4.6 days, *p* <.001) more than doubled during the pandemic year. For what concerns long-term effects of Covid-19 pandemic on ED healthcare services, in this study, authors report a significant difference in the pre-pandemic and pandemic phases (β= 1.941, t =3.673, *p*= .002), with a steep positive linear slope representing 68.2% of the increase of ED (which has continued in 2021). In alignment with the previous study, Ibeziako and colleagues reported a decrease in patients with public insurance (46.0% vs 41.7%, *p*=.008) and consequent increase in private insurance. In the retrospective study conducted by Otto et al. (23) on 248 children (mean age: 15.2 ± 2.6), counts of admissions for restrictive ED per month then increased significantly over time during the Covid-19 pandemic (β1 + β3 = 1.58 [95% CI: 1.25 to 1.91]; *p*<.001). The total number of admissions between April 1, 2020, and March 31, 2021, (n = 125) was more than double (123% increase) the mean number of admissions for the same time frame (April 1 through March 31) for the previous 3 years (mean = 56). Even in this study, significant change in the distribution of insurance types during the Covid-19 pandemic (*p* = .005), and the proportion of patients with public insurance decreased significantly (*p*= .02). Interestingly, authors report that limitations in in-person outpatient appointments were not a significant driver of the increase in admissions. Spigel et al. (10) aimed to explore the effects of the pandemic on the provision of ED-related care for adolescents and young adults. They examined the relationships between access to care, changes in outpatient care, perceived disruptions to care, and the quality of care, and the associations with ED thoughts and behaviors in a sample of 73 participants (n= 62 with a diagnosis of atypical anorexia) with a mean age of 19.1 ± 3. They conducted a retrospective study comparing data with pre-pandemic period (time frame investigated: June 2017 – February 2020). Findings from the study revealed that 81% of participants reported increased intrusive ED thoughts and behaviors after the onset of COVID-19. Additionally, 45% of the participants reported engaging in restrictive, compensatory, or binge-eating behaviors frequently or daily. Moreover, 47% of participants reported discontinuing care during the pandemic: specifically, 22% stopped mental health therapy, 10% stopped nutrition visits, and 32% stopped weight checks with physicians. Perceived treatment disruption was associated with lower quality of care (*p* = 0.004), higher odds of intrusive ED thoughts (adjusted odds ratio [aOR] = 2.63; 95% confidence interval [CI]: 0.56–12.3) and increased ED behaviors (aOR = 1.98; 95% CI: 0.63–6.19). However, access to care remained high, with 92% of respondents maintaining access to at least one provider via telehealth or in person. Among those with telehealth access (n = 64), 59% found care to be as good as usual. Maintained access to ED care was associated with higher odds of intrusive ED thoughts (aOR = 5.32; 95% CI: 0.72–39.6) but lower odds of increased ED behaviors (aOR = 0.67; 95% CI: 0.11–4.20). Springall et al. (12) also conducted a retrospective chart review in Australia involving 457 participants, with a primary ED diagnosis from 2017 to 2020. The majority of presentations reported in the pediatric hospital were new ED diagnoses, which increased of 90% each year. Notably, in 2020, ED presentations significantly exceeded the average number of presentations per year from 2017 to 2019 (N = 98.7, *p* = 0.01). Primary reasons reported for ED onset included social isolation and loneliness (32.3%), changes in routine leading to lack of motivation (25.6%), and factors like cessation of community sports, boredom, and increased focus on ED thoughts. A small percentage (3%) cited reduced food availability. A decline in new diagnoses of AN and atypical AN occurred between February and April 2020, followed by an increase in cases from May onwards, surpassing previous years’ records. For 40.4% of patients assessed in 2020, the onset of ED behaviors coincided with the lockdown, with 12.8% experiencing a relapse during this period. New cases of restrictive ED peaked in August 2020, but then stabilized and slightly decreased from September. Although there was a slight increase in the proportion of AN and atypical AN patients admitted to hospitals from 2017 to 2020 (55.9% to 62.4%), no significant changes in the incidence of purging behavior or suicidal/self-harm ideation across the studied years was reported (*p* = 0.85).

Hellner et al. (20), Levinson et al. (22) Peterson et al. (24) and Raykos et al. (25) investigated the effectiveness of telehealth format and transition to virtually delivered treatments due to Covid-19 restrictions.

In the beta trial conducted by Hellner et al. (20), two young women, aged 15 and 20 respectively, underwent 4-week of virtually delivered Family Based Treatment (FBT) between April and May 2020. Positive outcomes in terms of weight gain (at least 1 pound (lb)/0.5 kg per week over the course of the trial) and marked reduction of ED symptoms were reported, supporting the effectiveness of the virtually delivered FBT using and enhanced multidisciplinary care team. Both patients and carers positively perceived the virtually-based treatment and engagement and satisfaction were high. The study lacks follow-up periods. To test efficacy of a multidisciplinary intensive outpatient program delivered virtually via telehealth during the pandemic, Levinson et al. (22) conducted a between-groups study with 93 participants. Findings suggest no differences in outcomes via delivery mode, with the multidisciplinary telehealth ED program resulting in comparable outcomes to in-person treatment. Peterson et al. (24) conducted a pilot trial in the USA involving 12 children aged 2 to 7.5, primarily diagnosed with Avoidant/Restrictive Food Intake Disorder (ARFID; APA, 2) in 2020. Participants received outpatient follow-up either in-clinic or via telehealth. The study found that both groups met an equivalent percentage of their goals over time, with participants in the in-clinic group meeting 92% and those in the telehealth group meeting 100% of their goals. Findings therefore suggest that telehealth services may be equally effective as in-clinic services in achieving meaningful goals and maintaining appropriate participant and caregiver behavior during outpatient follow-up. In Australia, Raykos and colleagues (25) conducted a comparative observational study in Australia with 25 participants, with an average age of 24.2 years, diagnosed with AN, BN OSFED, and Unspecified Feeding or Eating Disorder (UFED; APA, 2) between March and April 2020. Despite the lockdown and transition to telehealth, substantial improvements were reported across all outcomes. Switching to telehealth led to reductions in ED symptoms and clinical impairment. Participants also reported improvements in mood (depression and anxiety). Furthermore, there was a significant improvement in BMI, returning from the underweight range to the typical weight range (*p* <.001, d = 2.93). Most patients (71%, n = 12) rated telehealth as being “as good as” or “better than” face-to-face consultations. Additionally, the quality of the therapeutic relationship during telehealth was rated “as good as usual” by 88% of participants (n = 15) compared to face-to-face interactions.

Overall, monocentric studies investigating the impact of Covid-19 pandemic on children and young adults with EDs revealed a concerning surge in ED-related hospital admissions, ranging between 56% and 188% (19, 23). This is accompanied by extended boarding periods, treatment disruptions and a shift in insurance types (10, 21). To address challenges such as limited availability, longer waitlists, barriers to direct transitions and treatment interruptions, bridge plans and telehealth-based multidisciplinary ED programs emerge as potential options. From studies included, telehealth services emerge as potentially equally effective as in-clinic services in achieving meaningful goals and maintaining appropriate participant and caregiver behavior during outpatient follow-up.

### Multicentric studies

Milliren et al. (26), Hartman-Munick et al. (29), Toulany et al. (30) and Vyver et al. (31) explored trends volumes of emergency department visits, hospitalization and outpatient ED care before, during and after the onset of Covid-19 pandemic across different public pediatric hospitals and ED specialist units. Monteleone et al. (27) and Monteleone et al. (28) provided also an outlook on barriers and facilitators in the pathway to specialist care.

In the retrospective study conducted by Milliren et al. (26) on 38 USA pediatric hospitals between January 2018 and June 2022, a total of 95% of hospitals had higher aggregate raw volumes and higher average monthly visit volumes in the post-onset period. In particular that meant that in the pre–COVID-19 period, the mean number of eating disorder-related admissions per hospital was 93.9 (SD = 116.6; range 3–514) compared with 150.8 (SD = 192.9; range 5–992) post–COVID-19 onset. After the onset of the pandemic, a higher proportion of inpatient admissions for eating disorders were among patients of adolescent age (14–17 years), female sex, white race, non-Hispanic, privately insured, and from higher-median income zip codes. In the second-year post-onset through the end of the study period (April 2021–June 2022), visit volume decreased over time (β= -6.3 per month; 95% CI: -9.0 to -3.5; *p*<.001). More diagnoses of AN were reported after the onset of Covid-19. ED inpatient admissions were slightly longer post-onset of the pandemic, which, in combination with increased volume, resulted in a nearly 66% increase in monthly average cumulative bed-days. Similarly, Toulany et al. (30) in their cross-sectional study collecting data from Ontario (Canada) public hospitals on emergency department visits and hospitalizations for EDs among children and adolescents (mean age: 10.1 ± 4.3), reported that these have significantly increased - 66% in children and 37% in adolescents, respectively - after the onset of the Covid-19 pandemic compared with pre-pandemic expected rates. Acute care visits increased immediately after the onset of the pandemic, reaching a 4-week peak annualized rate of 34.6 per 100,000 population (ED visits) and annualized rate of 43.2 per 100,000 population (hospitalizations) in October 2020. Acute care visits for EDs remained well above the 3-year pre-pandemic average through to December 26, 2020. Specifically, in children aged 3-13 ED visit rates were lower than expected in April and May before returning to expected levels in June, 2020 (aRR= 1.04, 95% CI = .66-1.63). However, such rates continued to be higher than expected before peaking in December, 2020 (aRR = 2.40, 95% CI = 2.08-2.77). Regarding hospitalizations, these were higher than expected after the onset of the pandemic (aRR = 1.29, 95% CI = 1.03-1.61). Among adolescents aged 14-17, both ED visits and hospitalizations were higher than expected for all the months after the onset of the pandemic (aRR = 1.43, 95% CI =1.33-1.53). To compare the number of adolescents and young adults seeking inpatient and outpatient care before and after Covid-19 onset, between January 2018 and December 2021, Hartman-Munick et al. (29) conducted a multicentric study across 14 USA hospital-based adolescent medicine centers and one nonhospital-based center, for a total of 3100 participants. They found a significant increase in both inpatient and outpatient eating disorder volume after onset of the pandemic that surpassed pre-pandemic patient care trends. With regards to inpatient admissions, authors found that the aggregate number of ED admissions across all sites reached 163 in December 2020, peaking at 208 in April 2021 with a mean of 181 per month through the last 8 months of 2021. Pooled results accounting for site showed that prior to the pandemic, inpatient ED-related admission volume was increasing by a mean of 0.7% (95% CI: 0.2%-1.3%) each month. In April 2020, there was a small, although not significant, immediate 14.6% decline (95% CI: −29.2% to 2.9%) on average in inpatient ED admissions followed by a significant 7.2% (95% CI: 4.8%-9.7%) per-month increase in volume on average through April 2021. Beginning in May 2021, there was an immediate, nonsignificant 15.5% decline (95% CI: −30.2% to 2.3%) in ED-related admissions followed by a significant decrease over time of 3.6% (95% CI: −6.0% to −1.1%) per month on average through December 2021. For what concerns outpatient admissions, the aggregate number of assessments across all sites was 274 in December 2020, peaking at 425 in March 2021, and there was a mean of 376 per month through the last 8 months of 2021. Immediately post-pandemic onset, outpatient ED assessment volume declined by 39.7% (95% CI: −50.4% to −26.7%) but then increased significantly over time by a mean of 8.1% (95% CI: 5.3%-11.1%) per month through April 2021. Starting in May 2021, there was a significant immediate decline of 22.0% (95% CI: −36.4% to −4.4%) in the number of assessments followed by a nonsignificant decrease over time of a mean of 1.5% (95% CI: −3.6% to 0.7%) per month through December 2021. Vyver et al. (31) conducted a hospital-based retrospective study in Canada involving two children’s hospitals, Alberta Children’s Hospital (ACH) and SickKids Hospital for Sick Children (SickKids), with a total of 1098 participants (mean age: 14 ± 1.96 and 14 ± 1.78 respectively) with data collected from March 2014 to March 2021. Most responsible diagnoses of AN were observed in 72.9% of children and adolescents from SickKids and 68.3% from ACH. Both hospitals showed similar patterns, indicating an increase in hospitalizations following the onset of the pandemic, with a 63% increase at SickKids and a 132% increase at ACH during the first year after the pandemic outbreak. However, a nonlinear pattern was observed in the final three months (December 2020 to March 2021) at each site, with lower counts suggesting fluctuations in hospitalizations during this period.

In their study, Monteleone et al. (27) conducted research across 16 Italian ED specialist units, encompassing regions from North, Center, and South Italy, involving a total of 312 participants diagnosed with EDs (AN=179, BN=63, BED=48, OSFED=22) from June 2020 onwards. Their main findings indicated that perceived therapeutic relationship quality had significant negative effects on general psychopathology (β=−0.16; *p*=0.007) and ED psychopathology worsening (β=−0.22; *p*=0.001). Conversely, heightened isolation and fear of contagion showed significant positive effects on general psychopathology worsening (β = 0.22; *p* = 0.002 and β = 0.16; *p* = 0.003, respectively) and ED psychopathology worsening (β=0.22; *p <*0.001 and β=0.17; *p*=0.002, respectively). Higher quality of perceived therapeutic relationship was associated with a lower increase in psychopathology severity, which, interestingly, was not significantly influenced by the type of treatment provided during the lockdown period. During the lockdown period, direct access to care was maintained for 2/3 of the sample, while the majority of the remaining sample received telehealth treatment. Monteleone et al. (28) conducted afterwards European multicentered study involving 11 ED specialist units in Italy, France, Spain, Czech Republic, Germany, Poland and UK to assess barriers and facilitators in the pathway to specialist care in Europe during Covid-19 pandemic. They found that longer time between the onset of symptoms of the current ED episode and access to a specialist ED unit was associated with higher age (*p*< 0.001), general psychiatric symptoms (*p*= 0.01) and low social class (*p*= 0.008). The suggestion to seek care that promoted being seen at the specialist ED unit came from patients themselves (42%) or family (40.3%) in most of the cases. Psychiatrists, general practitioners and psychologists were the most common health professionals who either started the specialist pathway to ED care or directly referred to ED specialized units. Median time elapsing between the onset of symptoms of the current ED episode and the access to a specialist ED unit was 2 years (Min=0; Max=36 months); the average number of health professionals included in the specialist pathway for ED care was 2 (Min=0; Max=10) and most of the participants (92%) did not directly access the specialist ED unit, but rather saw two other health professionals first. In the study authors highlighted also country-related differences: Spain, Czech Republic, and Germany were associated with delayed access to ED units in comparison to Italy, while UK had an earlier access.

Overall, the combined findings from included multicentric studies underscore the substantial impact of Covid-19 pandemic on pediatric and adolescents ED care. Post-pandemic onset, there was a marked increase in emergency department visits and hospitalizations, particularly for adolescents, females, and those from higher-income backgrounds. Despite findings revealed an overall significant rise in both inpatient and outpatient eating disorder care volumes, fluctuations in hospitalization trends also emerged in the final months post-pandemic (29, 31). Perceived quality of the therapeutic relationship and continuity of care delivery, rather than the specificity of treatment intervention, were identified as a potential protective factor against exacerbating of ED psychopathology (27). Additionally, Monteleone et al.’s European study identified barriers to accessing specialist care, including demographic factors and country-specific variations. These comprehensive findings collectively highlight the complex dynamics shaping pediatric eating disorder care during and after the pandemic, emphasizing the need for targeted interventions and healthcare system adaptations.

## Discussion

Findings from both monocentric and multicentric studies provide a broad perspective on the comprehensive impact of Covid-19 pandemic on pediatric and adolescent ED care, highlighting the alarming surge in ED-related hospital admissions and ED specialist visits and emphasizing the urgency for adaptive interventions and healthcare system modifications.

### Trends of ED admissions in emergency departments, inpatient and outpatient specialist care services

The current review reveals an exponential increase in pediatric hospitalization for youth with restrictive EDs, ranging between 56% and 188% (12, 19, 23, 31) during Covid-19. The average number of monthly admissions more than doubled, compared to the pre-pandemic period (21). Additionally, emergency department visits for EDs among children and adolescents increased significantly, particularly among children aged between 3 and 13 (30). For this group reported rates demonstrated a 66% increase after the onset of Covid-19 compared with pre-pandemic expected rates. A 37% increase in both ED visits and hospitalizations was reported in adolescents aged 14-17. Our findings are consistent with existing scientific literature on the subject (6, 32). Notably, the significant rise in hospitalizations correlates with an escalation in comorbid anxiety and depression among individuals with eating disorders, reaching up to 97% compared to the period before the onset of the Covid-19 pandemic (19). No significant changes in the incidence of purging behavior and suicidal or self-harm ideation were reported (12). Also, the number of contacts registered by national services, such as the Canadian National Eating Disorder Information Centre (NEDIC), were significantly higher (n = 439; χ^2^ = 92.74, *p* <.001) during the pandemic period compared to 2018 (n = 197) and 2019 (n = 312) (4). Telephone helplines of ED-related foundations also recorded a 57% increase in calls (12). Among the primary reasons for ED onset during the Covid-19 pandemic, the majority of individuals reported social isolation and loneliness as possible putative factors, followed by routine changes, lack of motivation and cessation of sport activity (12).

Regarding the long-term impact of Covid-19 on presentations in ED clinical services, new cases of restrictive ED peaked in August 2020, subsequently stabilized (12) and then declined in the second year post-onset in both inpatient and outpatient services (29), absolute visit volumes remained elevated relative to pre-pandemic levels (26). Changes and interruptions in ED care delivery during Covid-19 could additionally clarify the increase in hospitalizations (31), whereas the subsequent decrease in ED hospital admissions might be attributed to capacity issues, or an increased availability of telehealth strategies to avoid treatment disruptions and safe discharge plans. However, more structured support is necessary to establish efficient pathways to ED care, and prevent individuals from seeking care in acute and emergency department due to excessively long waiting lists for outpatient visits and the absence of public ED specialist outpatient services. After the onset of Covid-19, the majority of inpatients admissions were for AN and among female patients, non-Hispanic, privately insured and from higher-median income zip codes (26). However, this data may reflect lack of ED services able to provide accessible care for patients from lower social classes, who cannot be privately insured. The fact that the higher proportion of hospital emergency admissions for EDs were among patients suffering from AN, can be explained by the urgency for inpatient care deriving from somatic life-threatening comorbidities related to AN condition (33). In line with previous literature, the lack of access to care and treatment, coupled with social isolation, disruptions to routines, and the negative influence of media, has been suggested as a possible reason for the development of ED symptomatology and its exacerbation in those with pre-existing ED behaviors (4, 7, 12, 34–37). Therefore, this could partially account for the increase in seeking help in emergency departments.

### Barriers and facilitators to access specialist ED care

Regarding pathways toward specialist care for EDs, possible barriers and facilitators emerged especially during Covid-19 pandemic with the abrupt increase in ED cases (27, 28).

Several barriers to accessing specialist ED care were identified in their multicenter European study. One significant hindrance is the impact of socioeconomic status, particularly for individuals belonging to lower social classes, who face challenges in accessing ED units due to perceived treatment costs and inaccurate stereotypes associating EDs mainly with higher social classes. Cultural barriers, such as stigma, shame, and guilt, contribute to the reluctance of seeking specialized care, as well as personal factors, including denial, ambivalence, and poor health literacy, further impede access (38). Moreover, as suggested in the report by Herington and colleagues (38) structural challenges, like dismissive attitudes from primary care providers and disparities in referral patterns, affecting ethnic minorities, LGBTQ2S+ individuals, and non-binary individuals, add complexity to the issue. The role of others in prompting individuals to seek care is nuanced, with prompting from friends and workmates associated with delayed access to ED units, while prompting from family members and partners shortens access time. Country-specific variations, higher age, and low social class predicted delayed access to care, with a median time of two years elapsing between the onset of symptoms of the current ED episode and the access to a specialist ED unit (28).

On the other hand, the study highlighted several facilitators in accessing specialist ED care. Patients seeking specialized ED units often consulted psychiatrists, general practitioners, and psychologists, who played pivotal roles in initiating or directly referring to specialist ED care pathways. The involvement of general practitioners in accessing specialist care and ED services was not consistent across European countries. However, in countries like the UK, where this involvement was more common, the length of the ED pathway was reduced, ensuring earlier access to ED specialist care. Moreover, affective (depression, anxiety) and somatic symptoms were key drivers in activating the specialist pathway, becoming more pronounced upon referral to the ED unit. Recommendations to seek care at specialized ED units primarily came from patients themselves and their relatives. Family members and partners actively encouraged individuals throughout the ED treatment course, playing a crucial role as facilitators. The study suggests that the local organization of health care services plays a crucial role in facilitating early access to specialized ED treatment (28). Not only, having general practitioners who are attuned to and well-informed about EDs could streamline the process, minimizing the need for multiple consultations with different professionals and enabling prompt specialized intervention. Regardless of whether the treatment is conducted through telehealth or in-person, the perceived quality of therapeutic relationship has been identified as a crucial protective factor towards ED psychopathology deterioration (27). This suggests that ensuring continuity of care, irrespective of the mode of delivery, can serve as a preventive strategy to reduce relapse rates.

### Telehealth and bridge plans

Telehealth has emerged as a crucial component in the evolving landscape of ED care, especially in response to challenges posed by the Covid-19 pandemic. Emerging data and experiences indicate the feasibility of transitioning certain aspects of ED care, such as psychotherapy, to telehealth platforms, demonstrating the adaptability of virtual solutions in maintaining treatment continuity (34, 39, 40). However, it is emphasized that in-person medical evaluations remain critical for assessing various health parameters, especially somatic life-threatening conditions, and identifying signs necessitating hospital admission. Transitioning to virtual care/telehealth and decreased in-person medical attention might have therefore hindered the timely identification of illness symptoms and limited access to care, thereby impeding the prevention of weight loss and disease progression (31). The study by Couturier et al. (41) supports a strong recommendation for in-person medical evaluation when necessary, emphasizing the importance of equal access to treatment for marginalized groups. In this study, authors developed a list of good practice points for virtual care implementation and delivery to ensure efficacy and patient acceptability. In instances where it was necessary due to Covid-19 restrictions, the delivery of multidisciplinary and evidence-based treatment models through virtual means was well-received by both caregivers and patients (20, 25). This translated into a notable reduction in AN symptoms, with reported high levels of engagement and satisfaction. Studies focused on pediatric feeding disorders and dysphagia highlighted how telehealth offered a hybrid model for quality care when access to traditional in-person sessions was limited (42). This helped overcome service challenges due to limitations in service delivery, client accessibility, and family support resources. To face care delivery disruption, many ED hospital services rapidly switched to remote care, in order to ensure continuing accessibility to care services. This enabled clinicians to facilitate flexible appointment scheduling, minimize time and financial burdens associated with travel, promote treatment adherence, and lower the risk of Covid-19 transmission (43). Patients undergoing remote care have achieved significant improvements in ED symptoms and mood, with the magnitude of improvement comparable to historical benchmarks at the same clinic (25). Additionally, patients and caregivers have rated the quality of treatment and therapeutic alliance highly and quality resulted comparable to in-person care delivery in different ED groups (10, 24, 25, 43). However, remote care is not without limitations inherent to technology, such as challenges in monitoring vital signs, physical examinations, and weight, which may evoke anxiety among users reliant on such follow-up measures However, these studies provided no long-term follow-up measures, making it impossible to draw conclusions on the capacity of this protocol to maintain positive effects on ED symptomatology. A recent meta-analysis aiming to compare the efficacy of telepsychiatry and face-to-face treatment in different mental disorders (44) reported that face-to-face treatment was superior to telepsychiatry for eating disorders, suggesting that efficacy may vary according to disease type. In the field of EDs, it seems that switch to remote consultations and telepsychiatry during the pandemic period was well perceived by patients (45), however, due to the multidisciplinary nature of ED care, this transition resulted in other team members continuing to work in-person. Thus, multidisciplinary approaches in treatment and rehabilitation for EDs appear to be pivotal in ensuring positive clinical outcomes (46). In this approach, a team of professionals, including psychologists, therapists, dietitians, and prescribers, collaboratively addresses the complexities of EDs. This comprehensive model of ED care is adaptable across various levels, encompassing inpatient, residential, partial-hospital, intensive outpatient programs, and outpatient care settings (47, 48). Despite studies limitations, the fact that literature reports no significant differences between participants who received treatment via telehealth versus in-person format suggests that telehealth transition due to the Covid-19 pandemic can be effective for treatment delivery if multidisciplinary team-based approaches via telehealth are secured (10, 22, 24, 25).

Challenges associated with discharge planning during the pandemic, including limited availability and longer waitlists at ED treatment centers, have prompted the exploration of bridge plans as a potential solution (19). These plans offer a transitional option, allowing patients to be discharged home safely while awaiting higher levels of care, addressing barriers to direct transitions from hospitalization to ED treatment programs. Telehealth also emerges as a possible solution to secure a good quality of the therapeutic relationship and simultaneously contrast treatment disruptions, therefore preventing from ED psychopathology worsening in case of stressful events, such as Covid-19 pandemic (10, 24, 25, 27). The evolving landscape of telehealth and the integration of bridge plans demonstrate promising avenues for enhancing ED pathways to care, ensuring accessibility and continuity in the face of unprecedented challenges.

### Insurance shifts and challenges for ED care providers

Findings from 33,3% of the included articles (n= 3) highlighted a notable shift in the landscape of insurance coverage for individuals suffering from ED and seeking specialist care in the USA during the Covid-19 pandemic. There has been a significant increase in private insurance patients coupled with a decrease in government-based programs Medicaid patients compared to the pre-pandemic period (19, 21, 23). The distribution of insurance types underwent a significant change during the pandemic, emphasizing a decrease in patients with public insurance. Notably, the proportion of inpatient admissions for EDs shifted towards privately insured individuals, and those from higher-median income zip codes (26) suggesting challenges in accessing specialist ED care services through public healthcare avenues. In line with the position paper by Moreno and colleagues (49), the focus should be on retaining and enhancing existing mental health services and introducing new practices to extend access and deliver cost-effective care, particularly for those who developed mental disorders during the pandemic. In the field of EDs, ensuring multidisciplinary care service provision is crucial, refining current practices while acknowledging both the benefits and limitations of remote health delivery. Prioritizing accountability involves routine measurement of meaningful outcomes, co-production of service design and evaluation, expanding public health insurance coverage for mental health, and promoting greater integration of primary and secondary care (49). The Covid-19 pandemic warns against non-structured outpatient solutions that could worsen disparities in the provision of specialist ED care. The call is for a targeted, location-specific strategy to transform pandemic challenges into an opportunity to improve healthcare services for EDs. Specifically, there is a need for greater access to multidisciplinary care at the outpatient level, with a recommendation for utilizing telehealth as part of a bridge plan solution.

Overall, findings from included studies shed light on critical aspects within the ED healthcare landscape. The observed surge in acute hospital admissions for restrictive EDs such as AN - particularly when stratified by age - underscores the urgency for targeted interventions and resource allocation. The identified role of telemedicine emerges as pivotal in shaping hybrid models for patient management in ED care, emphasizing the potential benefits of technology integration in healthcare planning. Furthermore, the discernible shift towards private healthcare in response to ED care demand necessitates a comprehensive examination of healthcare delivery systems. This systematic review highlights the multifaceted nature of contemporary healthcare challenges and sets the stage for further exploration and refinement of strategies aimed at enhancing accessibility, efficiency, and the overall quality of specialist care for patients suffering from EDs.

## Limitations and future directions

The current systematic literature review emphasizes the significant challenges that Covid-19 has placed on mental health care systems. This includes addressing the heightened prevalence and incidence of EDs, an uptick in severe cases necessitating hospital admissions, and the resultant overcrowding of emergency departments. Covid-19 pandemic posed unprecedented challenges to health care systems globally. Consequently, some community hospitals redeployed staff to other overburdened hospitals or converted pediatric beds to adult care (31). This could potentially constrain the interpretation of data in included retrospective chart studies that compare and discuss pediatric hospital admissions. Among the limited number of included studies investigating these issues, a shortage of public outpatient multidisciplinary ED specialist services has surfaced, compelling individuals with EDs to transition to private insurance plans for specialized assistance. Concurrently, the studies also contemplate potential strategies, such as bridge plans and the implementation of telehealth, to address challenges arising from the pandemic. However, studies exhibit common limitations that could compromise the robustness and generalizability of their findings. Due to their monocentric nature, small sample sizes are a recurring issue in several studies (10, 19, 22–25), which may hinder the extrapolation of results to broader populations. Additionally, retrospective study designs, noted in Otto et al. (23), Ibeziako et al. (21), Spigel et al. (10) and Springall et al. (12) pose limitations as they rely on past data and may not account for real-time contextual factors or changes over time. Short observation period, non-validated measurement tools, and reliance on self-reporting, raise concerns about the reliability of findings across different studies included (10, 20, 24, 25, 27). Furthermore, the geographical distribution of the studies is skewed, with a significant majority (60%, n= 9) conducted in the USA, and only two multicentric studies in Europe (encompassing data from seven countries) and two in Canada: this could hinder the generalizability of results. Variations in geographical locations within a country and differing healthcare delivery regulations could also influence the observed disparities and outcomes across studies (12). Notably, there is a lack of information from other countries, especially those with lower welfare, limiting the global applicability of the findings. Most studies primarily focus on pediatric populations, and the investigation into the long-term effects of Covid-19 on the increase in ED cases and rates of admission is sparse, with only one study reporting data up to June 2022. Two studies, Toulany et al. (30) and Hartman-Minick et al. (29), do not provide specific diagnoses of EDs, making it impossible to draw conclusions about increasing rates of specific EDs. Additionally, few studies report the impact of Covid-19 on ED care delivery, providing data on the shift to virtual treatment, thereby reducing the generalizability of results and effectiveness. These also lack follow-up measures, limiting insights into the sustained effects of the such remote interventions implemented during the pandemic. Across studies, chart notes regarding the triggers for ED behaviors could also have reflected the expectations or biases of the clinicians (12, 27). These collective limitations underscore the need for cautious interpretation of results of the present literature review and highlight avenues for future research to address these gaps and enhance the overall understanding of the impact of Covid-19 on ED care delivery services. As from literature emerges ED symptoms deterioration during and after the onset of Covid-19 pandemic (10, 12, 19, 27), future research may address detailed strategies implemented by specialized ED units to address such symptom exacerbation as well as investigate sustained effects of adaptive interventions to enhance findings generalizability and develop guidelines for clinicians involved in ED care. A potential starting point could involve introducing innovative digital mental health practices, such as stepped-care models, in conjunction with preventive and self-management services alongside clinical care. This presents an opportunity to initiate change and address barriers, ultimately making these valuable digital services more widely accessible to individuals in need (50).

## Conclusions

The impact of Covid-19 on medical systems is widely acknowledged (51), however, the stress it imposed on mental health systems and treatment centers lacks comprehensive documentation (52, 53). This review article aimed to address this gap in the literature, focusing on the domain of EDs, which has experienced an unprecedented surge in hospitalizations and incidence rates during and after the pandemic period (6). Our findings from both monocentric and multicentric studies reveal a concerning rise in mental health challenges among youth, characterized by significantly increased trends in ED admissions during the pandemic, particularly regarding pediatric hospitalizations for restrictive EDs. The long-term impact on ED presentations remains elevated, indicating persistent challenges in accessing timely care. Barriers to specialist ED care, including socioeconomic status, cultural factors, and structural challenges, have been identified, necessitating targeted interventions. Telehealth emerges as a crucial component in the evolving landscape of ED care, demonstrating adaptability in maintaining treatment continuity. However, challenges in insurance coverage shifts and discharge planning underscore the need for comprehensive strategies to ensure accessibility and continuity in ED pathways to care. The findings emphasize the importance of multidisciplinary approaches, location-specific strategies, and the integration of telehealth as part of bridge plans to address the evolving challenges posed by the pandemic and improve healthcare services for individuals with EDs.

## Data availability statement

The original contributions presented in the study are included in the article. Further inquiries can be directed to the corresponding author.

## Author contributions

MB: Conceptualization, Data curation, Formal analysis, Methodology, Writing – original draft, Writing – review & editing. FB: Conceptualization, Data curation, Formal analysis, Methodology, Writing – review & editing. EA: Supervision, Validation, Writing – review & editing. LM: Conceptualization, Funding acquisition, Project administration, Supervision, Validation, Writing – review & editing.
